# Evaluation of endoscopic visible light spectroscopy: comparison with microvascular oxygen tension measurements in a porcine model

**DOI:** 10.1186/s12967-019-1802-x

**Published:** 2019-02-28

**Authors:** Rinse Ubbink, Louisa J. D. van Dijk, Desirée van Noord, Tanja Johannes, Patricia A. C. Specht, Marco J. Bruno, Egbert G. Mik

**Affiliations:** 1 Department of Anesthesiology, Laboratory for Experimental Anesthesiology, Erasmus MC University Medical Center, s Gravendijkwal 230, 3015 CE Rotterdam, The Netherlands; 2 Department of Gastroenterology and Hepatology, Erasmus MC University Medical Center, s Gravendijkwal 230, 3015 CE Rotterdam, The Netherlands; 3 Department of Radiology, Erasmus MC University Medical Center, s Gravendijkwal 230, 3015 CE Rotterdam, The Netherlands; 4 Department of Gastroenterology and Hepatology, Franciscus Gasthuis & Vlietland, Kleiweg 500, 3045 PM Rotterdam, The Netherlands; 5 Department of Intensive Care, Erasmus MC University Medical Center, Rotterdam, The Netherlands

**Keywords:** Visible light spectroscopy, Chronic mesenteric ischemia, Diagnostics, Microvascular oxygen tension measurements

## Abstract

**Background:**

Visible light spectroscopy (VLS) is a technique used to measure the mucosal oxygen saturation during upper gastrointestinal endoscopy to evaluate mucosal ischemia, however in vivo validation is lacking. We aimed to compare VLS measurements with a validated quantitative microvascular oxygen tension (μPO_2_) measurement technique.

**Methods:**

Simultaneous VLS measurements and μPO_2_ measurements were performed on the small intestine of five pigs. First, simultaneous measurements were performed at different FiO_2_ values (18%–100%). Thereafter, the influence of bile was assessed by comparing VLS measurements in the presence of bile and without bile. Finally, simultaneous VLS and μPO_2_ measurements were performed from the moment a lethal dose potassium chloride intravenously was injected.

**Results:**

In contrast to μPO_2_ values that increased with increasing FiO_2_, VLS values decreased. Both measurements correlated poorly with R^2^ = 0.39, intercept 18.5, slope 0.41 and a bias of − 16%. Furthermore, the presence of bile influenced VLS values significantly (median (IQR)) before bile application 57.5% (54.8–59.0%) versus median with bile mixture of the stomach 73.5% (66.8–85.8), p = < 2.2 * 10^−16^; median with bile mixture of small bowel 47.6% (41.8–50.8) versus median after bile removal 57.0% (54.7–58.6%), p = < 2.2 * 10^−16^). Finally, the VLS mucosal oxygen saturation values did not decrease towards a value of 0 in the first 25 min of asystole in contrast to the μPO_2_ values.

**Conclusions:**

These results suggest that VLS measures the mixed venous oxygen saturation rather than mucosal capillary hemoglobin oxygen saturation. Further research is needed to establish if the mixed venous compartment is optimal to assess gastrointestinal ischemia.

## Background

Visible light spectroscopy (VLS) is a technique used to measure the mucosal capillary hemoglobin oxygen saturation based on reflectance spectrophotometry [1]. The mucosal oxygen saturation can be calculated by the marked difference in the absorption spectra of oxygenated and deoxygenated hemoglobin. Endoscopic VLS measurements are performed during upper GI endoscopy [2–4]. As determined previously by van Noord et al., measurements are defined positive for ischemia if the measured saturation is lower than 63% in the antrum of the stomach, lower than 62% in the duodenal bulb and 58% in the descending duodenum [4].

VLS is used in clinical practice in the work-up of the diagnosis of chronic mesenteric ischemia (CMI). CMI is defined as ischemic symptoms caused by insufficient blood supply to the gastrointestinal (GI) tract [5]. The main cause of CMI is stenosis of one or more mesenteric arteries due to atherosclerosis [6]. Other occlusive causes are external compression of the celiac artery and/or celiac ganglion by the median arcuate ligament and diaphragmatic crura (median arcuate ligament syndrome (MALS)) and mesenteric artery stenosis due to vasculitis. However, CMI can exist in the absence of mesenteric artery stenosis. Non-occlusive mesenteric ischemia (NOMI) is caused by hypo-oxygenation due to underlying conditions such as cardiac and pulmonic insufficiency, spasms of small arteries, shunts, occlusion of smaller arteries, e.g. by micro-emboli, and autonomic dysfunction [7].

The diagnosis of CMI is a clinical challenge because of the diverse presentation of CMI. Symptoms overlap largely with many other disorders and the high prevalence of asymptomatic mesenteric artery stenosis in the general population of (3–29% [8, 9]) due to the existence of an extensive collateral circulation. However, mesenteric artery stenosis can become symptomatic if this collateral circulation is not sufficient and/or the extent of the stenosis becomes significant. Accurate identification of patients with CMI is important to select those patients who will benefit of therapy, but to withhold invasive therapy from those who will not. Treatment consists of endovascular revascularization with expandable metal stents or surgical revascularization of obstructed vessels, both methods that are invasive, costly and not without side-effects. A functional test to determine mucosal ischemia of the GI tract is therefore essential.

In the absence of one specific test for the diagnosis of CMI [10], the diagnosis is established by consensus in a multidisciplinary meeting attended by gastroenterologists, vascular surgeons and interventional radiologists. Symptoms alone do not accurately predict the diagnosis of CMI [7, 11, 12]. Therefore, consensus diagnosis is based on the combination of symptoms, imaging of the mesenteric vasculature and functional assessment of mucosal ischemia with gastric-jejunal tonometry [13, 14] or VLS [1, 4]. The diagnosis is confirmed if successful therapy results in symptom relief. This method for the diagnosis of CMI has an acceptable diagnostic yield [15] and this method is excepted in absence of a gold standard test [10].

Endoscopic mucosal oxygen saturation measurements with VLS are already used in clinical practice to evaluate CMI, however no extensive validation studies have been performed for this intended use. In the current study, VLS mucosal oxygen saturation is compared with a validated microvascular oxygen tension (μPO_2_) measurement technique [16, 17].

The microvascular oxygen tension technique used in this study is a Palladium (Pd) porphyrin phosphorescence lifetime technique that measures oxygen tension, introduced by Van der Kooi at the end of the 1980s [18]. Palladium porphine (Pd-porphyrin) bound to albumin, has become a standard phosphorescent dye for μPO_2_ measurements in vivo [16, 17]. This quantitative measurement is also located in the microcirculation making it a convenient comparison to mucosal oxygen saturations measured with VLS.

The objective of this study was to validate the VLS technique. This validation consisted of 3 experiments in a porcine model: (1) comparison of VLS mucosal oxygen saturation and μPO_2_ measurements at different levels of FiO_2_, (2) VLS mucosal oxygen saturation measurements in the presence of bile and (3) comparison of VLS mucosal oxygen saturation and μPO_2_ measurements during asystole.

## Methods

### Ethical statement

This study was approved by the local Animal Research Committee of the Erasmus MC University Medical Center in accordance with the National Guidelines for Animal Care and Handling (protocol number DEC 129-13-06 EMC3185). To enhance transparency this article is written according to the ARRIVE guidelines for animal research [19].

### Experimental animals

In total, 5 female crossbred Landrace x Yorkshire pigs, with mean body weights of 28.1 ± 0.6 kg (mean ± standard error of mean), age 2–3 months were used for the experiments. Sample size calculation determined that 5 animals were sufficient to detect a difference of at least 5% in mucosal saturation measured with VLS before and after bile per location with an alpha of 0.05 and a power of 90% [20].

### Experimental procedures

After an overnight fast with free access to water, the animals were sedated with an intramuscular injection of tiletamine/zolazepam (6/6 mg/kg; Virbac Laboratories, Carros, France), xylazine (2 mg/kg; AST Farma B.V., The Netherlands) and atropine sulfate (0.5 mg/animal; Centrafarm Services BV, Etten-Leur, The Netherlands). After a 15 min induction period, anesthesia was induced with tilatamine/zolazepam (50–100 mg/animal) through a cannula (20G Venflon (Becton, Dickinson and Company, USA) in an auricular vein. Tracheal intubation was performed with a size 7.0 Portex^®^ endotracheal tube (Smiths Medical International Ltd., United Kingdom). For maintenance of anesthesia, the animals received continuous infusion of ketamine (5 mg kg^−1^ h^−1^; Alfasan Nederland B.V., The Netherlands), midazolam (1.5 mg kg^−1^ h^−1^; Atavis Group PCT, Iceland), sufentanil (4 μg kg^−1^ h^−1^; Janssen-Cilag B.V., The Netherlands), and rocuroniumbromide (4 mg kg^−1^ h^−1^; Fresenius Kabi Austria GmbH, Austria). All animals received 500 ml of colloid solution (Voluven^®^; Fresenius Kabi AG, Germany) at start and a continuous infusion of crystalloid (Sterofundin^®^ ISO 10 ml kg^−1^ h^−1^; B. Braun, Germany). Each pig received a bolus of magnesium sulfate (500 mg; Pharmachemie BV, Haarlem, The Netherlands), as arrhythmia prophylaxis, added to the first bag of crystalloid solution. To prevent infections during the experiment, Cefazolin (1000 mg/animal; Kefzol ^®^ EuroCept BV, Ankeveen, The Netherlands), an antibiotic used for the treatment of a widespread of bacteria was given intravenous.

Pressure-controlled mechanical ventilation (Servo 300; Siemens-Elema, Solna, Sweden) was performed with a FiO_2_ between 24% and a positive end-expiratory pressure of 5 cm H_2_O while no intervention was done. Normothermia, measured nasal, was maintained between 38 and 39 °C, with two heating pads underneath and an electric heating blanket above the animal. Furthermore, hearth rate, MAP, SpO_2_ and temperature were monitored continuously throughout the entire experiment. Arterial blood samples were collected to determine the arterial oxygen pressure and arterial oxygen saturation (ABL 800Flex (Radiometer, Denmark).

A 4F thermodilution catheter (Pulsion Medical Systems AG München, Germany) was placed in the left femoral artery for arterial blood sampling. An 9Fr introducer sheath (Arrow International Inc., USA) was placed in the right jugular vein for infusion of palladium porphyrin. Both catheters were placed using the Seldinger technique. A lower midline abdominal incision was made to insert a cystostomy tube into the urinary bladder with purse-string sutures for urine collection.

**Fig. 1:**
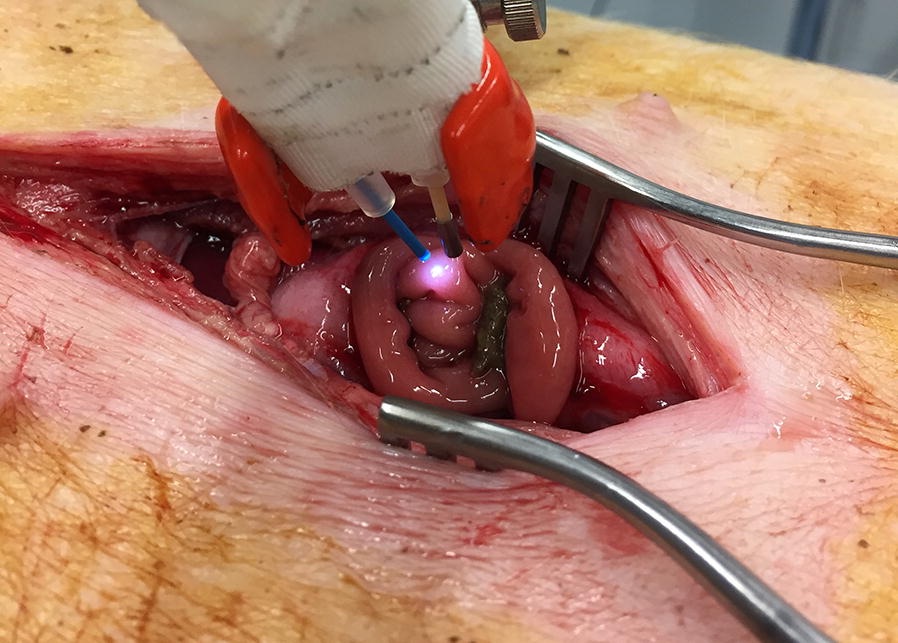
Set-up of the experiment of the VLS-probe (blue) and the μPO_2_ probe fixated together positioned 1 to 5 mm above the mucosa of the small intestinal loop

The animals were placed in supine position and an incision was made to open the abdomen. A small intestinal loop was dissected and a small incision was made at the non-vascularized side to expose the intestinal mucosa (Fig. 1). Mucosal oxygen saturation measurements were performed with a fiberoptic probe (Endoscopic T-Stat Sensor; Spectros, Portola Valley, California, USA) connected to the VLS oximeter (T-Stat 303 Microvascular Oximeter, Spectros, Portola Valley, California).

Microvascular oxygen tension measurements were done with oxygen dependent phosphorescent dye palladium porphine (Pd-porphyrin). Palladium porphyrin is a large molecule with optical properties that can absorb energy and react with oxygen. In the absence of oxygen it will release the absorbed energy from an excitation source via phosphorescent light with a specific decay time, i.e. lifetime. The lifetime is related to the amount of oxygen surrounding the Pd-porphyrin described by the Stern–Volmer relation [18]. It has been tested for pH, temperature and diffusivity dependency [17]. Calibration experiments are done and determine the O_2_ accuracy of 5% independent of phosphorescence intensity itself [17].

For the laboratory experimental setup of the μPO_2_ measurements the excitation source was an Opolette 355-I tunable laser (Opotek, Carlsbad, CA, USA) set to a wavelength of 524 nm. An optical fiber developed by TNO and produced by Light Guide Optics was used that would fit through the working channel of a gastroduodenal endoscope. It has one central located excitation fiber with several surrounding detection fibers.

The phosphorescence was collected with a gated micro channel plate photomultiplier tube (MCP-PMT R5916U series, Hamamatsu Photonics, Hamamatsu, Japan). Phosphorescence lifetime analysis was done with a self-written software program in Labview (version 13.0, National Instruments, Austin, TX, USA). For the detailed setup description we refer elsewhere [21].

The probe palladium porphyrin was Pd(II) meso-Tetra (4-carboxyphenyl)porphine (80 mg/animal) (Frontier Scientific, Logan, USA) dissolved in 1 ml DMSO and TRIS Trisma^®^ Base (Sigma, St. Louis, MO) was combined with a 4% bovine serum albumin solution solved in phosphate buffered saline. This method has been validated in vitro and in vivo [17]. Pd-porphyrin bound to albumin, forms a high-molecular-weight complex, confining it mainly to the vascular compartment when infused intravenously.

Both optical fibers were fixated together to perform stable simultaneous mucosal oxygen saturation and μPO_2_ measurements of the same mucosal spot of the small intestine (Fig. 1).

### Mucosal oxygen saturation versus μPO_2_ measurements at different FiO_2_ values

**Fig. 2:**
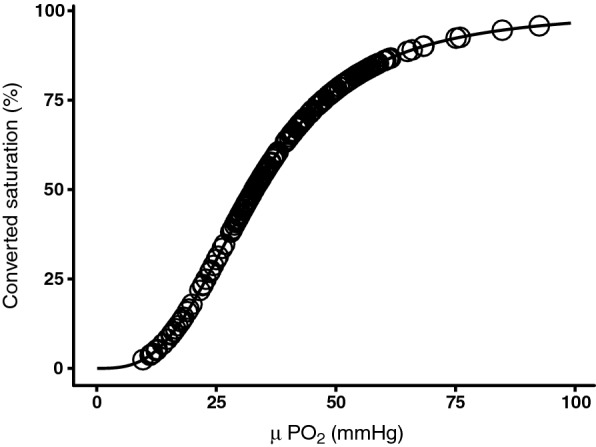
Conversion of μPO_2_ into saturation according to the found relationship by Serianni et al. [23]

Simultaneous VLS mucosal oxygen saturation and μPO_2_ measurements were performed at different FiO_2_ values ranging from 18 to 100%. The mucosal oxygen saturation and μPO_2_ measurements were simultaneously performed for 2 min at a specific FiO_2_ value. When a new FiO_2_ value was set, the start of a set of new measurements was awaited for the first two 2 min. To compare the two measurement techniques the μPO_2_ was converted into a corresponding saturation. For the μPO_2_ conversion, for every measured value in mmHg the corresponding % was calculated called micro-vascular oxygen saturation converted (μSO_2_.converted). The conversion can be found in Fig. 2.

### Influence of bile on mucosal oxygen saturation

Furthermore, the influence of bile on mucosal oxygen saturation values measured with VLS was assessed. Mucosal oxygen saturation measurements were performed of the small intestine mucosa in presence of bile. Two different types of bile were used: fluid obtained during upper GI endoscopy from the stomach of the animal and fluid obtained from the small intestine of the animal. The sticky viscosity of the bile ensured the fixation of the bile on the measurement area and continuous visual confirmation ensured that the bile measurements were performed on surface covered with bile. The amount of bile applied to the mucosa, the thickness of the bile applied and the exact content of the bile applied were not controlled. The mucosal oxygen saturations in presence of bile were compared with the mucosal oxygen saturations before the bile was applied to the mucosa (baseline) and the mucosal oxygen saturations every time after the bile was removed with saline fluid as control. For every step approximately 30 measurements were done.

### Mucosal oxygen saturation versus μPO_2_ during asystole

Finally, simultaneous mucosal oxygen saturation and μPO_2_ measurements were performed from the moment a lethal dose potassium chloride was intravenously injected. A measurement period of 25 min after injection was considered long enough to ensure a steady state since Benaron et al. showed detection of local ischemia with VLS within 120 s [22].

### Experimental outcomes

Mucosal oxygen saturation values were defined in percentage tissue hemoglobin saturation. The μPO_2_ measurements were defined in mmHg.

### Analytical and statistical methods

Statistical analysis was performed with R Statistics software (v3.2.4). Normal distribution was assessed visually and with the Shapiro–Wilk normality test. Normal distributed data is presented as mean ± standard deviation (SD) and abnormally distributed data is presented as median with interquartile range (IQR). A linear regression model was used for the FiO_2_, mucosal oxygen saturations, and μPO_2_. A scatter plot was used to show the mucosal oxygen saturation versus the μPO_2_ measurements at different FiO_2_ values. To compare the two measurement techniques, the μPO_2_ was converted from mmHg to % porcine hemoglobin saturation. To determine the saturation a porcine-specific hemoglobin saturation formula published by Serianni et al. was used [23]: (%/100) = (0.13534 × P_O2_)^3.02^/[(0.13534 − P_O2_)^3.02^ + 91.2]. The formula was derived from 213 data point at pH 7.4 and 37° with an excellent fit.

To compare the saturation, the difference in measurement frequency had to be overcome. The mucosal oxygen saturation has a fixed measurement interval whereas the μPO_2_ is measured on demand. To equally compare the two measurements the mucosal oxygen saturation was averaged over same period as one μPO_2_ was done. Thereafter these results were visualized with linear regression and with a Bland–Altman comparison plot [24].

The Wilcoxon signed-rank test was used to compare the measurement before, with and after application of bile. A two-tailed p value of < 0.05 was considered significant. After the potassium chloride injection mucosal oxygen saturation measurements were compared with μPO_2_. Because VLS measures every second, a symmetrical moving average of 20 samples was taken to smooth the data, for example the eleventh sample is an average of sample [1–21].

## Results

### Baseline data

All 5 animals were in good clinical condition before the start of the experiment.

### Mucosal oxygen saturation versus μPO_2_ measurements at different FiO_2_ values

**Fig. 3:**
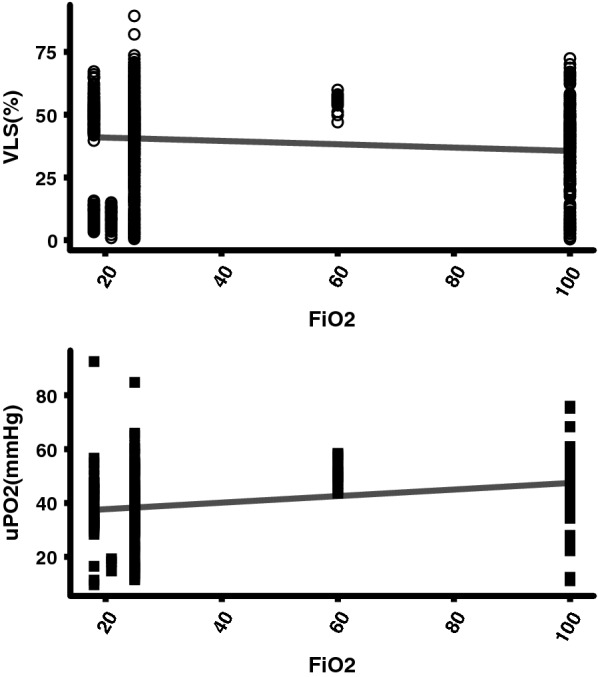
Scatter plot n = 5 of mucosal oxygen saturation versus μPO_2_ measurements at different FiO_2_ values. VLS (R^2^ = − 0.01, Intersect = 42.19, Slope = − 0.07), μPO_2_ (R^2^ = 0.06, intersect = 35.56, slope = 0.14)

The mucosal oxygen saturation levels versus the μPO_2_ levels different values of FiO_2_ in 5 animals were measured. The mucosal oxygen saturation decreased with increasing FiO_2_ in contrast to the μPO_2_ values that increased with increasing FiO_2_. The spread of the mucosal oxygen saturation levels and the FiO_2_ levels was large, shown in Fig. 3.

**Fig. 4:**
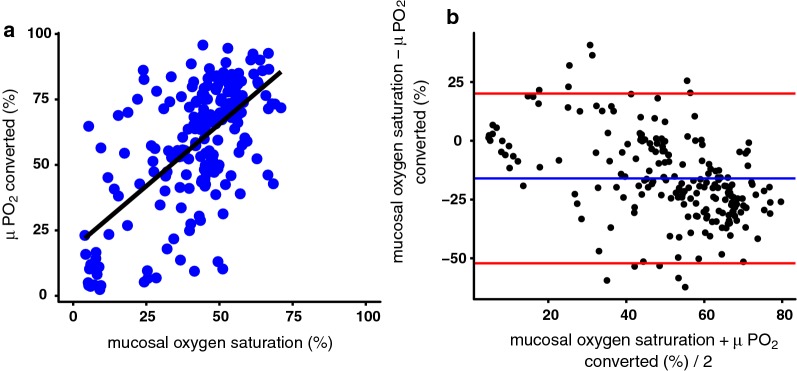
**a** Correlation between mucosal oxygen saturation and the converted μPO_2_ saturation. R^2^ = 0.39, intercept 18.5 slope 0.41. **b** Blant–Altman plot of the mucosal oxygen saturation and the converted μPO_2_ saturation. VLS—μPO_2__saturation: − 16.00974, 2.5% limit: − 52.83358, 97.5% limit: 20.81410, SD (diff): 18.41192

Figure 4a shows the correlation between mucosal oxygen saturation and the converted μPO_2_ saturation. There is a poor linear correlation with an r^2^ = 0.39, an interception of 18.5% and a slope of 0.41. In the Bland–Altman plot (Fig. 4b) also a poor correlation is seen with a mean difference of − 16%. If the saturation increases the mucosal oxygen saturation undervalues the saturation even more.

### Influence of bile on mucosal oxygen saturation

**Fig. 5:**
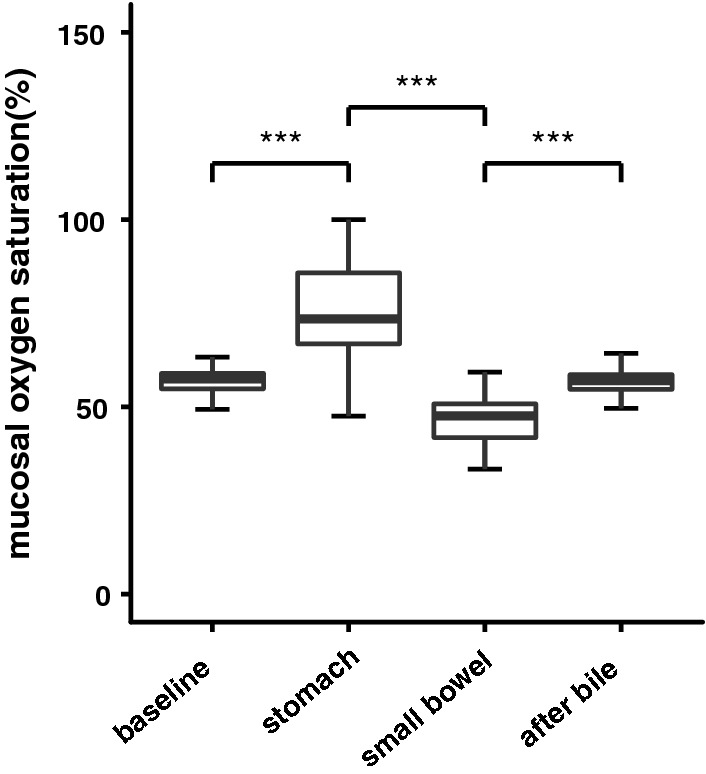
Mucosal oxygen saturation measurements without the presence of bile, with the presence of a bile mixture from the stomach, with a bile mixture from the small bowel and measurements without any of the bile mixtures. The baseline mucosal oxygen saturations did not significantly differ from the mucosal oxygen saturations after the bile had been removed shown as “after bile”. ***p < 2.2 * 10^−16^

Figure 5 shows the mucosal oxygen saturation measurements without the presence of bile, with the presence of a bile mixture from the stomach and with the presence of a bile mixture from the small bowel and measurements without any of the bile mixtures measured in a total of 2 animals. The mucosal oxygen saturation measurements before application of the bile mixtures and after the bile mixtures were removed were not significantly different (mucosal oxygen saturation before application of bile mixture median (IQR) 57.5% (54.8–59.0%) versus mucosal oxygen saturation after removal bile mixture 57.0% (54.7–58.6%), p = 0.2743). However, a significant increase of the mucosal oxygen saturation was seen when the bile mixture from the stomach was applied compared to the mucosal oxygen saturation before application of the bile mixtures (median mucosal oxygen saturation with mixture of the stomach (IQR) 73.5% (66.8–85.8) p = < 2.2 * 10^−16^). When the bile mixture from the small bowel was applied, the mucosal oxygen saturation was significantly lower with a median (IQR) 47.6% (41.8–50.8), p = < 2.2 * 10^−16^ compared to mucosal oxygen saturation measurements with bile mixture form the stomach and the mucosal oxygen saturation increased significantly after the bile mixtures had been removed (p = < 2.2 * 10^−16^).

### Mucosal oxygen saturation versus μPO_2_ during asystole

**Fig. 6:**
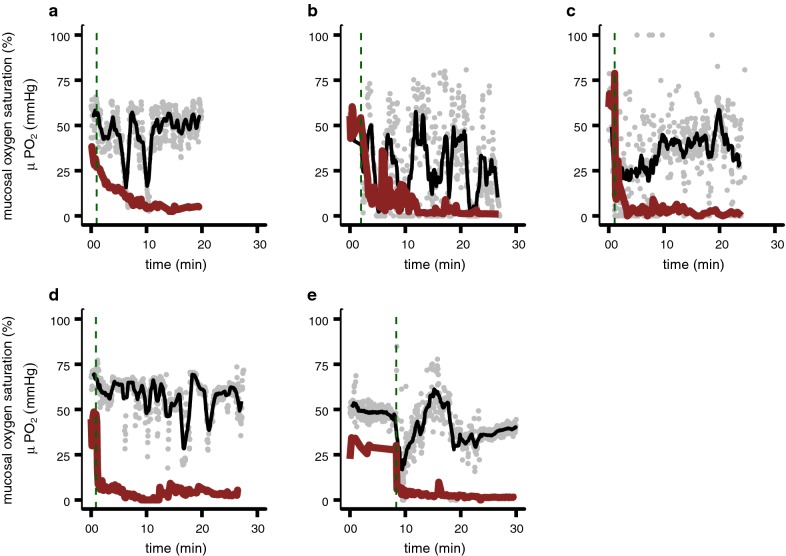
Average mucosal oxygen saturation measurements measured by VLS (black) over 21 data points (gray) and μPO_2_ measurements (red) during the minimally first 20 min of asystole in 5 pigs. Green vertical dashed line represents the time a lethal potassium dose was injected

The mucosal oxygen saturation measurements and μPO_2_ measurements during the minimally first 25 min of asystole in 5 animals are shown in Fig. 6. In all 5 animals the μPO_2_ measurements decreased towards a value of 0. The mucosal oxygen saturation measured with VLS decreased and increased variably during the measurement period and the mucosal oxygen saturation never reached a stable state around 0%.

### Adverse events

No adverse events occurred during the 5 porcine experiments.

## Discussion

In this study we validated mucosal oxygen saturation measurements by comparing VLS with calibrated μPO_2_ measurements. This study showed that the mucosal oxygen saturation values decreased with increasing FiO_2_ in contrast to the μPO_2_ values that increased with increasing FiO_2_ with a large spread of the measured mucosal oxygen saturation levels and FiO_2_ levels and a poor linear correlation. Furthermore, a significant influence of bile on the mucosal oxygen saturation values was shown. Finally, this study showed that the mucosal oxygen saturation values, in contrast to the μPO_2_ values, did not decrease towards a value of 0 in the first 25 min of asystole.

The found inverse relationship of the mucosal oxygen saturation measurements by VLS with FiO_2_ is remarkable. Mucosal oxygen saturations measured with VLS are expected to increase with increasing FiO_2_ if VLS measures the capillary oxygen saturation level. However, VLS measures not only arterial saturation but also a large venous compartment. If a large mixed venous saturation determines the overall saturation value the influence of FiO_2_ is expected to be minimal. Potentially due to hyperoxic vasoconstriction the actual venous saturation can decrease more compared to normoxic situations. The high FiO_2_ values will be measured by the μPO_2_. Furthermore, the measured values, both VLS as μPO_2_ values, have a great spread. Possibly, the oxygen tension was very variable in the gastrointestinal vessels as intestinal ischemia is also patchy and heterogenic distributed [5]. During the experiment the hemodynamic state of the animals worsened by all experimental handlings, also contributing to a great spread of measured values.

Significant influence of bile on the mucosal oxygen saturation values measured with VLS was confirmed. Therefore it is advised and mentioned in the prescription to remove any bile remnants before the start of the VLS measurements. The bile has its own absorption spectrum of light. It also absorbs light in the same wavelengths as oxyhemoglobin and deoxyhemoglobin [25], and influences the result to determine the mucosal oxygen saturation. The amount of bile applied to the mucosa, the thickness of the bile applied and the exact content of the bile applied were not controlled in this experiment. However, these factors contribute to the light absorption by the bile and thus influence its effects on the VLS signal. Therefore, we advise to remove any fluid on the measuring area of the GI mucosa before the VLS measurements.

The idea that VLS measures mixed venous oxygen saturation is further confirmed by the fact that VLS measured still a reasonable oxygen saturation 25 min after asystole. The saturation in the capillaries is decreased towards zero over time due to diffusion of oxygen towards the still oxygen consuming cells. However, in the venous compartment the oxygen will desaturate slowly by the large buffer capacity. Therefore, the oxygen saturation will not decrease towards zero immediately after asystole. Dips in oxygen saturation are seen in the mixed venous compartment measured by VLS as shown in Fig. 6 due to spasm in the supplying arteries. After such a peristaltic contraction the blood flow stabilizes and no decrease in saturation is seen.

VLS is a powerful technique to measure oxygen saturation at a microvascular level. In the microvasculature oxyhemoglobin/deoxyhemoglobin is proportional mainly located in the venous compartment of the microvasculature. Therefore the saturation measured by the VLS is mainly represented by the venous compartment. For detection of an oxygen transport problem that results in ischemia, the microvascular arterial saturation is of importance, a part that is underexposed by VLS. This is endorsed by the fact that after a lethal potassium chloride the saturation does not drop in comparison to μPO_2_, which is an exaggerated model of instant ischemia.

This study has some limitations. First, the experiments performed in this study were designed to enable generalizability in humans. However, to enable stable oxygen saturation measurements with VLS and μPO_2_ of the mucosa of the small intestine of a pig, the abdomen had to be opened to open the small intestinal loop. The mucosa of this small intestinal loop was exposed to room air and room temperature. This will result in oxygen diffusion into the tissue and rapid decrease in temperature for the exposed tissue. Furthermore, the abdominal anatomy of a pig is different from the human abdominal anatomy. The GI tract of a pig is monogastric like the human GI tract, however the colon lies in a spiral. The mesenteric vascularization in humans consists of individual variable, mesenteric vessel formations with arcades, lateral branches and anastomoses in the bowel wall [26]. The mesenteric vascularization in pigs consists of bundles of vessel branched of the main stem arising from the mesentery and passing directly into the bowel wall without any branching of arcades [26].

## Conclusion

This study showed that VLS measures the mixed venous hemoglobin oxygen saturation and not the mucosal capillary hemoglobin oxygen saturation. The presence of bile significantly influences the oxygen saturation levels measured with VLS. VLS is currently used in clinical practice in the clinical work-up of CMI. Further research is needed to establish if the mixed venous compartment is optimal for mucosal hemoglobin saturation measurements to assess GI ischemia.

## Abbreviations

CVP: central venous pressure
CCO: continuous cardiac output
CMI: chronic mesenteric ischemia
GI: gastrointestinal
IQR: interquartile range
MALS: median arcuate ligament syndrome
MAP: mean arterial blood pressure
μPO_2_: microvascular oxygen tension
NOMI: non-occlusive mesenteric ischemia
Pd: palladium
SD: standard deviation
SVV: stroke volume variability
VLS: visible light spectroscopy
